# Neonatal Episodic Hypoglycemia: A Finding of Valproic Acid Withdrawal

**DOI:** 10.4274/jcrpe.v2i2.92

**Published:** 2010-05-09

**Authors:** Dilek Çoban, Selim Kurtoğlu, Mustafa Ali Akın, Mustafa Akçakuş, Tamer Güneş

**Affiliations:** 1 Erciyes University Faculty of Medicine, Department of Pediatrics, Division of Neonatology, Kayseri, Turkey; +90 352 437 49 37+90 352 437 58 25drdilekcoban@yahoo.com.trErciyes University Faculty of Medicine, Department of Pediatrics, Division of Neonatology, 38039 Kayseri, Turkey

**Keywords:** newborn, hypoglycemia, valproic acid, withdrawal

## Abstract

The treatment of epilepsy during pregnancy is a worldwide problem. Drugs need to be used to control seizures in the mothers. In utero, exposure to valproic acid (VPA) and phenytoin (PH) may cause congenital malformations and also withdrawal symptoms such as irritability, jitteriness and symptoms of hypoglycemia. We present here a newborn with episodic hypoglycemia due to in utero exposure to VPA and PH. The mother was diagnosed as having complex partial epilepsy and was treated with PH (200 mg/day) and VPA (600 mg/day). The offspring developed jitteriness on the second day of life. The infant was hypoglycemic (32 mg/dl). These findings were accepted as withdrawal symptoms, since serum levels of VPA and PH were 37.8 μg/ml (50−100 μg/ml) and 6.37 μg/dl (10−20 μg/ml), respectively. Measurement of blood glucose is important and should be carefully monitored in infants exposed to antiepileptics in utero.

**Conflict of interest:**None declared.

## INTRODUCTION

Epilepsy is common and has a prevalence of 5.25 per 1000. One third of epilepsy patients are women of reproductive age. Most women with epilepsy require ongoing antiepileptic drug (AED) therapy during pregnancy. Both seizures during pregnancy and AED exposure in utero are thought to affect the poor outcomes seen in babies born to epileptic mothers (1). In utero, AED exposure may cause congenital malformations and may also lead to withdrawal symptoms in the newborn (1, 2, 3, 4, 5). We present here a newborn patient with withdrawal symptoms due to valproic acid (VPA) and phenytoin (PH) exposure in utero.

## CASE REPORT

A male infant was born at 35 weeks gestation by caesarean section because of placenta previa. He had bradycardia and difficulty in breathing, requiring resuscitation. The 21−year−old mother had complex partial epilepsy and was treated with PH (200 mg/day) and VPA (600 mg/day). The patient was the third child of non−consanguineous parents. The mother had undergone three abortions. The baby’s weight was 2720 g, length 56 cm, head circumference 36.5 cm. Physical examination revealed cyanosis, intercostal retractions, tachypnea, and hypotonia. Laboratory findings were as follows: Hemoglobin: 18.9 g/dL, WBC: 20800 mm^3^/L, PLT: 114 000 mm^3^/L, AST: 139 IU/L (N: 0−40), ALT: 34 IU/L (N: 0−40), CPK:3233 IU/L (N: 40−226 IU/L), CPKMB:380 IU/L (N: 2−20 IU/L) LDH: 2152 IU/L (N: 100−190 IU/L). Other laboratory studies, including blood glucose levels, did not reveal any abnormality.

In our intensive care unit, the patient was monitorised and head box oxygen and fluid repletion were initiated. His vital functions normalized, but he developed jitteriness on the second day of life. Laboratory investigations revealed hypoglycemia (32 mg/dL) and IV glucose infusion was initiated. Because his mother took VPA and PH for her epilepsy, the baby was evaluated for withdrawal symptoms and blood drug levels were analyzed. VPA level was 37.8 μg/mL (normal levels 50−100 μg/mL) and PH level was 6.37 μg/dL (normal levels10−20 μg/mL) and were measurable, although none of these were used in the offspring. Following the IV glucose infusion, the blood glucose level became normal and the jitteriness disappeared. Oral feeding was started with gradual reduction of the infusion rate. Hypoglycemia was observed again on the sixth day (38 mg/dL). IV treatment was continued until the 10^th^ day of life, when oral feedings were fully initiated. Hypoglycemia did not recur and the patient was discharged.

## DISCUSSION

The optimal management of epileptic women during their childbearing years and, particularly during their pregnancies, presents a considerable clinical dilemma. It has been recognized that treatment with AED during pregnancy is related to an increased risk for major congenital malformation. However, the justification for treatment has been that the risks of seizures during pregnancy are greater than those of AED therapy (1). Maternal death rate is approximately 10 times higher in women with epilepsy than in the general population. The death histories in the reports include seizure occurrence often associated with stopping AED or with poor compliance. There is little data regarding risk for malformations or other adverse outcome in the fetus as a result of seizures (6). Major and minor congenital anomalies have been reported to occur with most AEDs, including carbamazepine, PH, and VPA (7).

A number of adverse effects, including teratogenesis, liver toxicity and weight gain have been reported with VPA treatment (2, 4, 5, 6, 7, 8, 9). Long−term VPA treatment can cause carnitine deficiency (2, 10). VPA is 80 to 90% bound to plasma proteins. VPA crosses the placenta and is found in higher concentrations in cord serum than in maternal serum (2, 11). It is metabolized by the liver (2). Nau et al (12) showed that VPA is excreted in the newborns with a mean half−life of 47±15 hours, this duration being approximately 4 times the mean value found in adult epileptics. Meador et al (13) showed that the highest overall incidence of malformations occur in pregnancies exposed to VPA. When AEDs were compared in monotherapy groups, VPA continued to show the highest incidence of malformed births.

The most frequent major congenital malformations occurring with AEDs are neural tube defects, congenital heart defects, oral clefts, genital abnormalities, and limb defects (2, 5, 14, 15). In fetuses exposed to VPA, neural tube defects occur 10 times higher than the normal incidence and appear to be specifically associated with VPA therapy rather than with other antiepileptics (2). Effects on major organ systems are expected to occur with first trimester exposure and with maternal doses of over 1000 mg a day (2, 8, 14, 15). More recently there has been concern about a possible increased risk of neurodevelopmental problems in children with VPA exposure. Neurodevelopmental effects are more likely to be due to exposure later in the pregnancy, when brain cells are proliferating and migrating. Developmental milestones are often delayed in these children, particularly in the area of speech. Learning and behavioral abnormalities show a higher incidence in these children compared to normal children (1, 6, 14). Koch et al (16) reported cerebral dysfunction in children exposed to VPA in utero. Our patient’s mother was on 600 mg/day VPA and 200 mg/day PH for five years. Our patient will be followed for any neurodevelopmental aberrations.

PH is another drug widely used for different types of epilepsy. It is metabolized in the liver. In utero, exposure to PH may lead to congenital anomalies which are described as the fetal hydantoin syndrome. This syndrome includes microcephaly, growth retardation, dysmorphic face, orofacial clefts, cardiac defects and distal digital hypoplasia with small nails (3, 8, 12). Although exposed to PH in utero, our patient did not have any of these abnormalities.

Withdrawal signs of AED are also very important in the neonatal period. In newborns, the signs of withdrawal are irritability, jitteriness, abnormal tone, seizures, feeding problems and hypoglycemia. The signs occur at birth (3, 4, 8, 15, 17). Our patient had jitteriness and hypoglycemia. Hypoglycemia appears during the first few hours after birth, but may also occur in the first few days (4). Usually hypoglycemia is detected between 12 and 48 hours after birth and is dose−related (2, 15) Hypoglycemia in these babies is more common than in infants of diabetic mothers (4). Ebbesen et al (4) have suggested that hypoglycemia can be episodic. During episodes of hypoglycemia, ketone bodies originate from fatty acids and may be an alternative substrate for cerebral metabolism, but VPA inhibits this ketogenesis. Thus, hypoglycemia in infants exposed to VPA in utero may be hypoketotic. Additionally, VPA inhibits gluconeogenesis and mitochondrial ß−oxidation of fatty acids. Thus, hypoglycemia in VPA exposed infants results from decreased gluconeogenesis and reduced liver glycogen and/or impaired glycogenolysis (4). Transient mild elevations of ALT, AST and LDH were found in children on VPA monotherapy (18, 19). Both VPA and PH may increase serum AST, ALT, and LDH levels (20). Impaired psychomotor development is frequently seen in infants exposed to VPA in utero. Unrecognized hypoglycemia may will be one of the reasons leading to impaired psychomotor development in these children (4). PH exposure in utero may cause withdrawal symptoms, but it generally induces hyperglycemia by inhibiting the release of insulin as a result of the blockage of calcium uptake via voltage−dependent calcium channels (21). We detected two cases of hypoglycemia in PH−exposed infants in the relevant literature. The attempt to escape from the inhibitory effects of PH on insulin secretion or the increased sensitivity of the tissues to insulin were suggested as possible mechanisms leading to hypoglycemia (21, 22). However, hypoglycemia is a rare manifestation of PH exposure and VPA is more potent than PH in carbohydrate metabolism.

In conclusion, infants exposed to VPA have greater risk for hypoglycemia during the first week and especially in the first two days of life. Repeated episodes of hypoglycemia in these infants are reported in the literature. We recommend that blood glucose should be carefully monitorized in these infants, particularly in the first two days after birth.
